# Evaluating micro-computed tomography for investigation of the pediatric hyoid-larynx complex

**DOI:** 10.1007/s00247-025-06364-6

**Published:** 2025-08-14

**Authors:** GMM Timmerman, A Van Goethem, D Docter, J Hagoort, Y Dawood, NHJ Lobe, QD Gunst, MJB Van Den Hoff, HM De Bakker, RR Gorter, W Jacobs, RR Van Rijn, V Soerdjbalie-Maikoe, BS De Bakker

**Affiliations:** 1 Department of Obstetrics and Gynecology, Amsterdam University Medical Centers, Meibergdreef 9, Amsterdam, 1105 AZ Netherlands; 2 Department of Forensic Medicine and Pathology, Antwerp University Hospital, Antwerp, Belgium; 3 Department of Pediatric Surgery, Amsterdam University Medical Centers, Amsterdam, Netherlands; 4 Amsterdam Gastroenterology Endocrinology Metabolism, Amsterdam, Netherlands; 5 Amsterdam Reproduction and Development research institute, Amsterdam, Netherlands; 6 Department of Medical Biology, Amsterdam UMC Location AMC, Amsterdam, Netherlands; 7 Department of Radiology and Nuclear Medicine, Amsterdam University Medical Centers, Amsterdam, Netherlands; 8 Department of Radiology, Groene Hart Hospital, Gouda, Netherlands; 9 Department of Pediatric Surgery, Erasmus MC – Sophia Children’s Hospital, University Medical Center Rotterdam, Rotterdam, Netherlands

**Keywords:** Diffusible iodine-based contrast-enhanced computed tomography, Forensic imaging, Histology, Hyoid-larynx complex, Micro-computed tomography, Pediatrics

## Abstract

**Background:**

Conventional CT imaging has limitations in detecting subtle fractures or soft tissue hemorrhages of the pediatric hyoid-larynx complex due to its largely unossified, cartilaginous structure, creating a diagnostic gap in forensic investigations.

**Objective:**

To explore the feasibility of micro-computed tomography (micro-CT) and diffusible iodine-based contrast-enhanced computed tomography (diceCT) as high-resolution imaging techniques for detailed forensic and developmental assessment of the pediatric hyoid-larynx complex.

**Materials and methods:**

Five pediatric hyoid-larynx complex samples were obtained during forensic autopsies. Specimens were excised, fixed in formaldehyde, and scanned using micro-CT. Subsequently, samples were stained with buffered Lugol’s solution and rescanned for diceCT. Imaging was performed with voxel sizes between 12–35 µm. Scans were assessed by a trained analyst and two experienced forensic (pediatric) radiologists.

**Results:**

All five samples were successfully imaged using micro-CT and diceCT. Ossification increased with age: the youngest sample showed minimal ossification, while the oldest showed ossification of the hyoid lesser horn and thyroid cartilage. Anatomical variants included bilateral triticeal cartilages and ossified stylohyoid ligament fragments. DiceCT enabled detailed soft tissue visualization and revealed hyperdense bundles and ossification centers within the thyroid cartilage. Staining was complete in smaller samples but limited in the largest. No traumatic injuries were detected on imaging or autopsy.

**Conclusion:**

Micro-CT and diceCT offer high-resolution visualization of both ossified and soft tissue structures in the pediatric hyoid-larynx complex. These methods overcome limitations of conventional CT, demonstrating strong potential to enhance forensic evaluation of pediatric neck trauma.

**Graphical Abstract:**

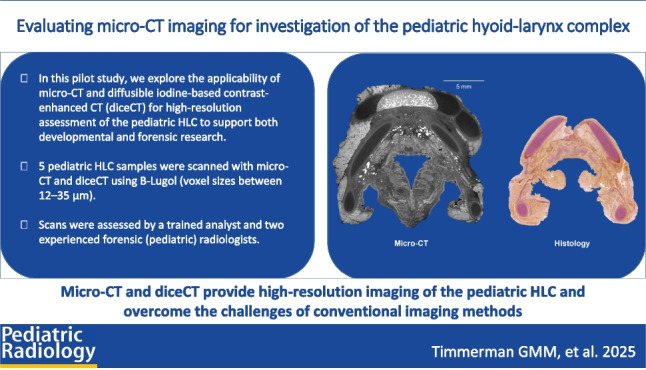

## Introduction

In forensic practice, evaluation of the hyoid-larynx complex is pivotal in diagnosing fatal neck trauma, including strangulation and choking [1, 2]. Fractures of the hyoid-larynx complex provide critical evidence for trauma-related causes of death. However, imaging the pediatric hyoid-larynx complex poses unique challenges due to its lack of calcification [3, 4]. Standard forensic imaging methods, such as total-body CT, struggle to resolve the soft tissue structures and cartilaginous components, leading to the potential misdiagnosis of fractures and hemorrhages [5, 6]. Furthermore, anatomical variants within the hyoid-larynx complex can mimic fracture patterns, increasing the risk of false-positive interpretation [3]. Histological analysis, while the gold standard for fracture assessment and the differentiation between ante- and postmortem injuries [5], is time-consuming, destructive to the specimen, limited to two-dimensional sections, and subject to sampling error due to the inability to analyze the entire sample [7]. Consequently, there is a need for alternative, non-destructive imaging techniques that can provide detailed anatomical information, particularly in pediatric cases.

Micro-computed tomography (micro-CT) is a high-resolution imaging modality that has gained traction in biomedical research [8]. While based on the same principles of X-ray attenuation as conventional CT, micro-CT allows for substantially higher spatial resolution [9, 10]. Unlike clinical CT scanners, micro-CT systems typically employ a stationary X-ray source and detector, with the sample being rotated during imaging. This setup permits a shorter source-sample distance and a longer sample-detector distance, resulting in greater magnification and isotropic voxel sizes in the micrometer (µm) range [9]. This high level of detail could make micro-CT particularly useful in forensic research, where precise visualization of skeletal and cartilaginous structures is necessary and ex vivo research is possible. To enhance soft tissue visualization, diffusible iodine-based contrast-enhanced computed tomography (diceCT) is utilized [11]. This technique involves submerging tissue samples in Lugol’s iodine (I_2_KI), a water-based iodine solution that passively diffuses into tissues [11]. Due to its strong affinity for blood, Lugol’s iodine highlights hemorrhagic areas as hyperdense regions on imaging, thus facilitating the distinction between antemortem injuries, which typically involve hemorrhage, and postmortem fractures, which do not [12, 13]. The diagnostic value of combining micro-CT and diceCT for evaluating trauma to the hyoid-larynx complex has already been demonstrated in adult forensic cases, where the technique enabled detection of subtle fractures and hemorrhages that were missed by conventional imaging or autopsy [14].

**Fig. 1:**
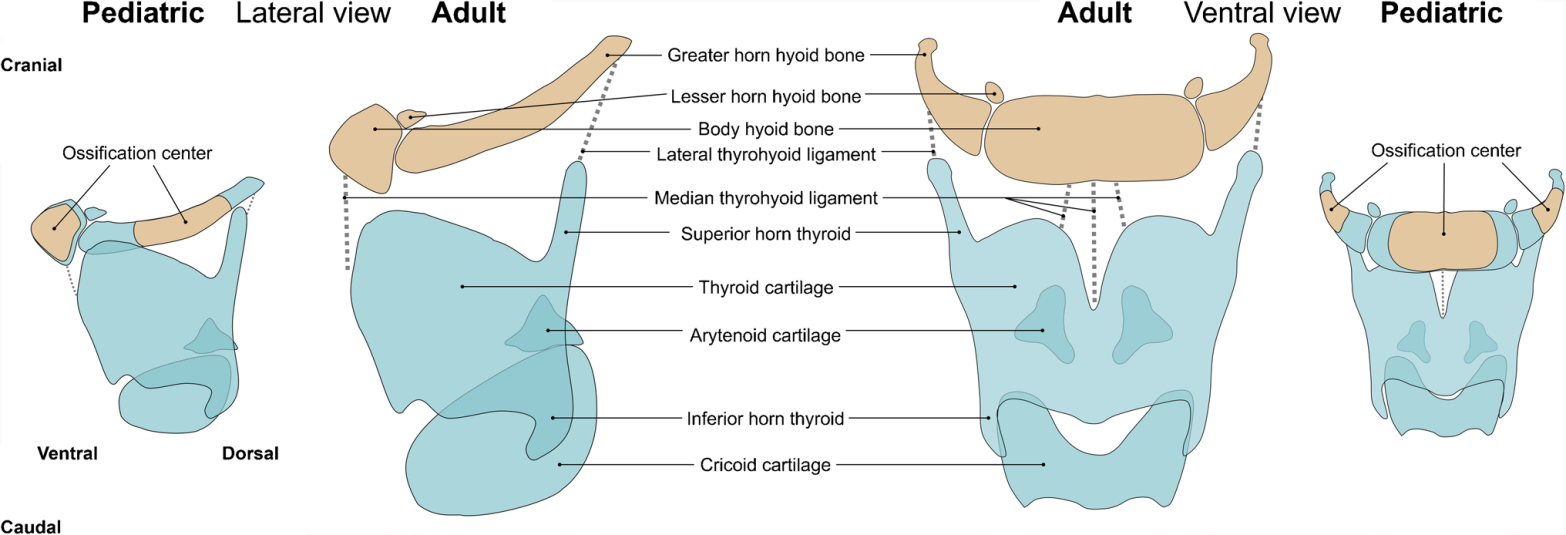
Overview of the normal pediatric and adult hyoid-larynx complex anatomy. The left side of the image shows the lateral view and the right side illustrates the ventral view of both the pediatric and adult hyoid-larynx complex. Anatomical structures are labeled in the adult hyoid-larynx complex, while ossification centers are indicated in the pediatric hyoid-larynx complex

Beyond trauma assessment, this imaging approach also offers opportunities for detailed anatomical investigation, particularly in studying developmental changes and anatomical variation across age groups. The hyoid-larynx complex undergoes significant morphological changes during growth and maturation. Figure 1 illustrates the normal anatomy of the pediatric hyoid-larynx complex in comparison to the adult hyoid-larynx complex. Within the hyoid bone, the connections between the central body and the greater horns may progressively ossify with age, leading to partial or complete fusion, observed bilaterally in approximately 25% of adults and unilaterally in 10–12% [3, 15]. Variation is also common in the lesser horns, which may differ in size, show fusion, or even be absent [3, 16]. As development continues into adolescence, the laryngeal cartilages begin to calcify, typically starting in the inferior horns of the thyroid cartilage. This process advances with age and differs between sexes [16, 17]. In children, however, these structures remain largely cartilaginous, influencing their biomechanical behavior and susceptibility to injury or deformation. Additional variability can be found in the presence of a separate triticeal cartilage within the lateral thyrohyoid ligament, seen in 8–30% of adults but not well documented in children, likely due to delayed ossification during adolescence [3, 15]. Notably, these small cartilages may be mistaken for fractures of the superior thyroid horn on imaging.

Given these complex and dynamic developmental changes, a comprehensive understanding of pediatric hyoid-larynx complex anatomy is essential for accurate forensic interpretation and clinical assessment. In this pilot study, we aim to evaluate the applicability of micro-CT and diceCT for high-resolution analysis of the pediatric hyoid-larynx complex to support both forensic diagnostics and developmental anatomical research.

## Materials and methods

**Fig. 2:**
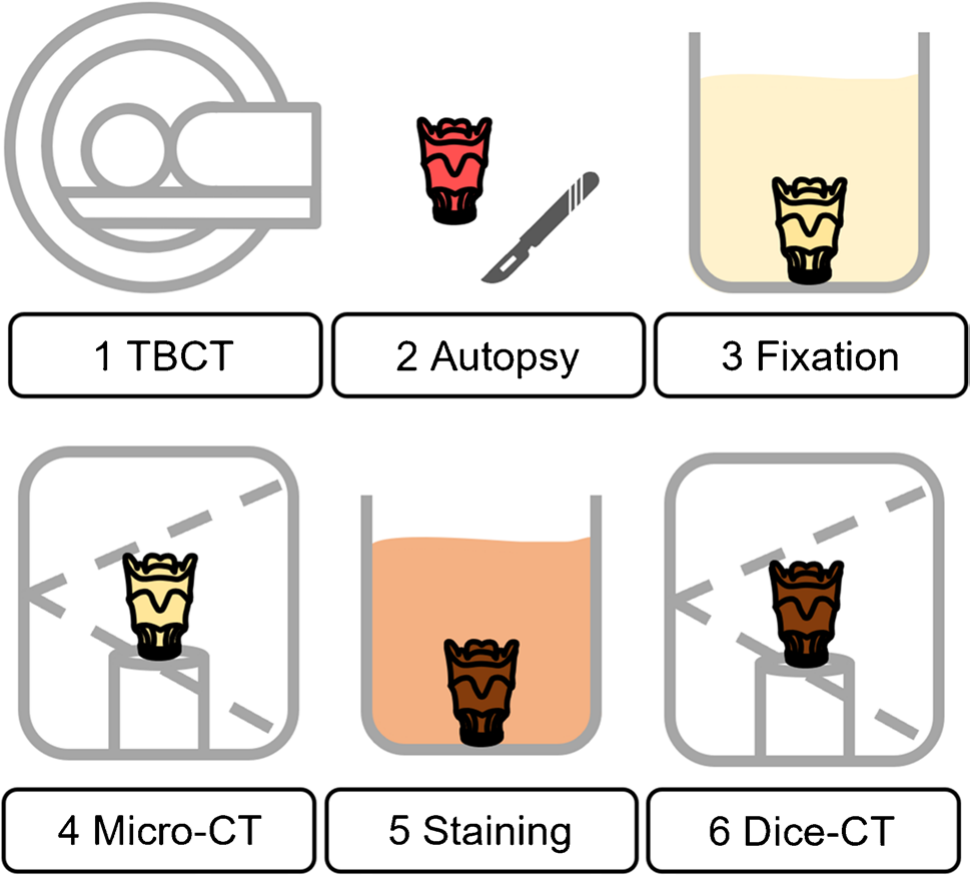
Methods workflow. (1) Postmortem total-body CT as part of the forensic examination. (2) Autopsy with excision of the hyoid-larynx complex. (3) Fixation of the sample with 4% formaldehyde. (4) Micro-CT scanning of the included specimen. (5) Staining of the samples by submersion in 3.75% B-Lugol. (6) DiceCT scanning of the stained samples. Adapted from Timmerman et al. [14]

The methodological workflow, presented in chronological order, is depicted in Fig. 2.

### Sample acquisition

Five pediatric hyoid-larynx complex samples, ranging from 12 days to 16 years of age, were obtained from forensic autopsies (February–August 2022). Based on the preliminary information provided at the time of case referral, suspected neck trauma was considered unlikely in three cases, deemed possible in one case, and was suspected in another case (Table 1).

**Table 1:** Sample characteristics. Case number; sex: male or female (M/F); age in days (d), months (m), or years (yr); context of death; and if there was any suspicion of trauma to the neck prior to the autopsy

Case (*n*)	Sex	Age (yr)	Cause of death	Suspected neck trauma
1	F	0 (12 d)	Sudden unexpected infant death (SUID)	No
2	F	1 (20 m)	Foreign body aspiration	No
3	M	5	Traffic accident	Possible
4	M	14	Found decomposed in bed	No
5	M	16	Traffic accident	Yes

During the autopsy, the on-duty forensic pathologist (including V.S.M.) excised the hyoid-larynx complex in accordance with local standardized procedures. This process required an incision in the neck, followed by the removal of the superficial throat muscles and thyroid gland. The hyoid-larynx complex was then carefully dissected, along with the tongue and the upper section of the trachea. Once isolated, the tongue was separated, allowing for a manual assessment of the stability of the hyoid bone and superior thyroid horns. Each excised hyoid-larynx complex, together with the surrounding soft tissue if present, was subsequently preserved in 4% formaldehyde.

### Sample preparation

Before imaging, the samples were thoroughly rinsed with phosphate-buffered saline (PBS) solution and placed in small padded plastic containers to ensure stability during scanning.

In order to perform diceCT, the samples were stained. Traditional Lugol’s solution is acidic and can cause tissue shrinkage [18]. To counter this effect, a buffered variant, Buffered Lugol’s solution (B-Lugol), has been developed in our lab to reduce tissue shrinkage while preserving staining effectiveness [18]. Contrast enhancement was achieved by submerging the specimens in 3.75% B-Lugol, using a solution volume equivalent to 20 times the weight of the sample (w/v). Based on our previous experiences and the hyoid-larynx complex’s weight, the staining process lasted between 4 and 16 days (Table 2).

**Table 2:** Stain parameters

Sample (*n*)	Weight (g)	Duration (d)	Amount 3.75% B-lugol (L)
1	4	4	0.1
2	10	5	0.2
3	17	5	0.3
4	43	12	0.9
5	121	16	2.4

### Imaging protocols

Micro-CT and diceCT imaging were performed by a trained micro-CT technician (D.D.) using the Phoenix Nanotom M (GE Sensing & Inspection Technologies GmbH, Pforzheim, Germany). For the largest sample, a different micro-CT system, the TESCAN UniTOM XL (TESCAN, Brno, Czech Republic), which is equipped with a higher-powered radiation source, was used. The samples were scanned at voxel sizes between 12 µm and 35 µm. A detailed overview of the scan parameters is presented in Table 3.

**Table 3:** Micro-CT and diceCT scan parameters. The GE Phoenix Nanotom M and TESCAN UniTOM XL were used for micro- and diceCT scanning. “Sample number-Lugol” indicates a stained sample scanned with diceCT. Parameters include scan time (h:mm); voxel size (μm); voltage (kV); current (μA) or power (W), depending on the scanner type; number of projections (*n*); aluminum (Al) filter thickness (mm); and exposure time (ms)

Sample (*n*)	Scan time (h:mm)	Voxel size (µm)	Voltage (kV)	Current (µA) or power (W)	Projections (*n*)	Filter (mm)	Exposure time (ms)
GE Phoenix Nanotom M							
1	1:05	12	60	400 µA	1875	0.5 Al	500
1-Lugol	1:05	12	60	350 µA	1875	0.5 Al	500
2	1:20	15	60	250 µA	2250	0.5 Al	500
2-Lugol	1:22	15	80	300 µA	2300	0.2 Al	500
3	0:51	25	60	250 µA	1500	0.5 Al	500
3-Lugol	0:45	25	100	250 µA	1350	0.5 Al	500
4	0:59	30	80	300 µA	1725	0.5 Al	500
4-Lugol	0:50	30	90	350 µA	1500	0.5 Al	500
TESCAN UniTOM XL							
5	0:28	35	80	35 W	2500	1.0 Al	235
5-Lugol	0:30	35	110	35 W	2500	1.0 Al	235

Standard forensic assessment for pediatric cases includes total-body CT, which was performed in all cases prior to the autopsy. The total-body CT imaging was done using a clinical CT scanner, Revolution CT (GE Healthcare, Chicago, IL) and included a protocol with voxel sizes of 625 µm. The scans were assessed by one of two clinical radiologists with experience in forensic imaging. Scans and reports were retrieved to compare with the micro-CT and diceCT scan results.

### Data analysis

Images were processed using Amira Software version 3D 2021.2, which was also used for visualization and 3D volume rendering (Thermo Fisher Scientific, Waltham, MA). Anatomical variations, ossification stages, potential fractures or hemorrhages, and notable hyper- or hypodensities were assessed by a trained analyst (G.M.M.T.) and two experienced forensic (pediatric) radiologists (H.M.D.B. and R.R.V.R.). These assessments were performed visually. Because micro-CT data are presented in arbitrary grayscale units rather than standardized Hounsfield Units (HU), interpretation relied on relative contrast differences between tissues. This allowed differentiation between mineralized and non-mineralized components, as well as detection of subtle density variations within cartilage.

## Results

The five samples were successfully scanned with micro-CT and diceCT.

### Ossification and anatomical variants

**Fig. 3:**
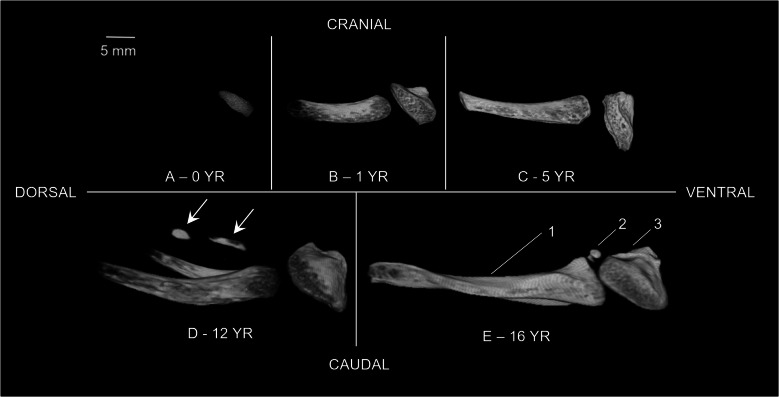
Ossification and anatomical variants. Lateral right side view 3D volume rendering models of blank micro-CT scans with voxel sizes between 12 μm and 35 μm, window adjusted to show only the ossified parts of the hyoid-larynx complex. (**a**) The sample of the 0-year-old female with only an ossified hyoid body (case 1) (12 μm). (**b**) The sample of the 1-year-old female with an almost complete ossified hyoid body and greater horns with non-fusion of the greater horns and cartilaginous lesser horns (case 2) (15 μm). (**c**) The sample of the 5-year-old male with a complete ossified hyoid body and greater horns with non-fusion of the greater horns and cartilaginous lesser horns (case 3) (25 μm). (**d**) The sample of the 12-year-old male with an ossified hyoid body and greater horns with non-fusion of the greater horns, cartilaginous lesser horns and as anatomical variant two bone fragments in the stylohyoid ligament (*arrows*) (case 4) (30 μm). (**e**) The sample of the 16-year-old male with an ossified hyoid bone with non-fusion of the greater horns and an ossified lesser horn on the right side and a cartilaginous lesser horn on the left (case 5) (35 μm). (1) Greater horn of the hyoid. (2) Lesser horn of the hyoid. (3) Hyoid body

**Fig. 4:**
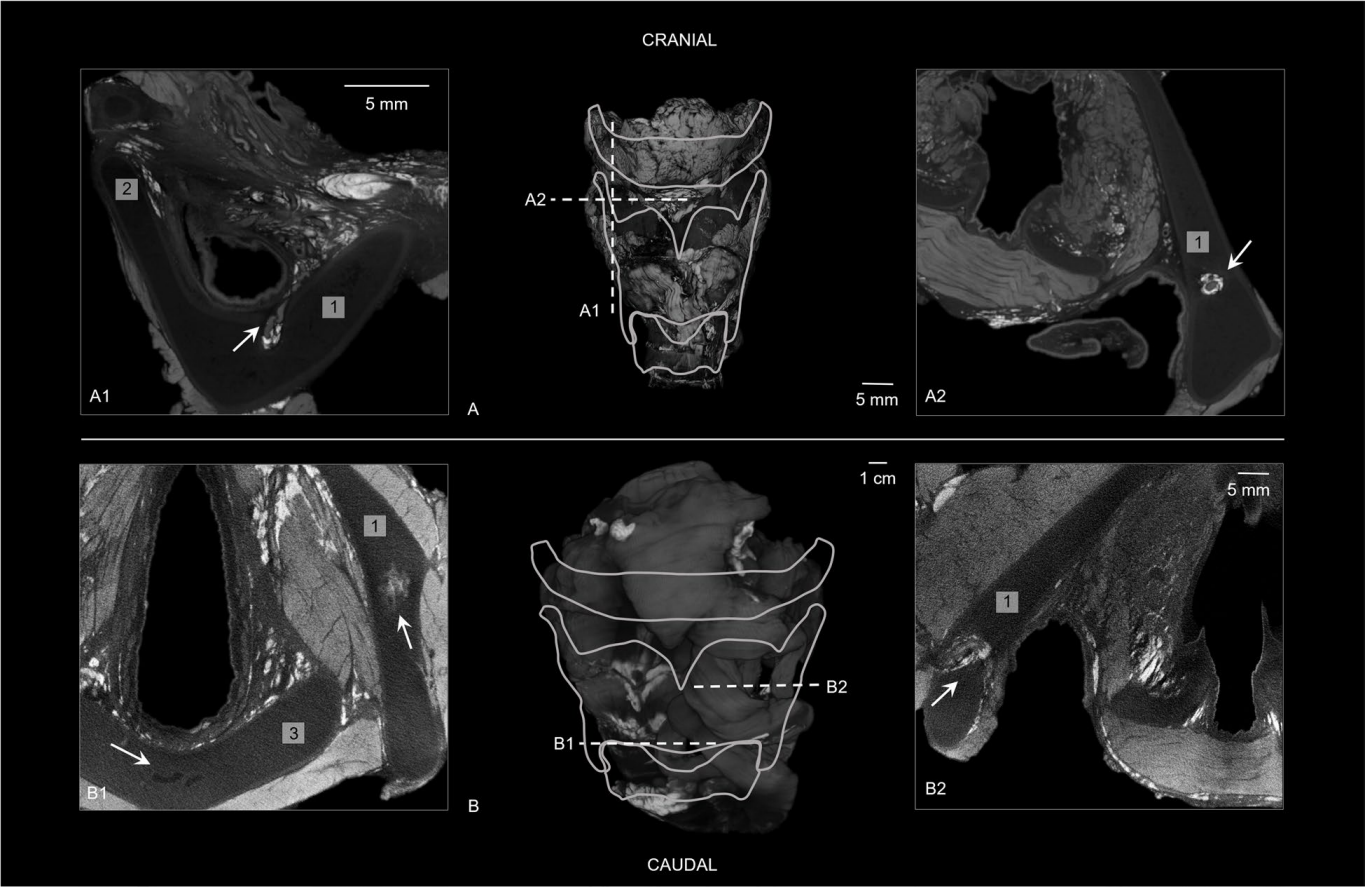
Thyroid cartilage on diceCT. (**a**) Frontal view of 3D diceCT volume rendering indicating sections (a1) and (a2) (case 3: 5-year-old male) (25 μm). (a1) Sagittal diceCT section showing a hyperdense bundle (*arrow*) crossing through the right lamina of the thyroid and a line with higher density was noted encircling the hypodense thyroid cartilage. The opening in the thyroid is also known as the foramen thyroideum. (a2) Transverse diceCT section of the hyperdense bundle with hypodense content (*arrow*) and a line with higher density was noted encircling the hypodense thyroid cartilage. (**b**) Frontal view of 3D diceCT volume rendering indicating sections (b1) and (b2) (case 5: 16-year-old male) (35 μm). (b1) Transverse diceCT section of the right lamina of the thyroid and the cricoid cartilage, showing a hyperdense ossification center in the thyroid cartilage (*right arrow*) and a few hypodense aspects in the cricoid cartilage (*left arrow*). (b2) Transverse diceCT section showing a hyperdense bundle with hyper- and hypodense content (*arrow*), crossing through the left lamina of the thyroid. (1) Thyroid cartilage. (2) Superior thyroid horn. (3) Cricoid cartilage

All samples exhibited incomplete ossification on micro-CT imaging (Fig. 3). The youngest sample (12 days old) showed ossification limited to the hyoid body, with cartilaginous greater and lesser horns of the hyoid and laryngeal structures. The other samples showed (almost) complete ossification of the hyoid body and greater horns. Only the oldest sample showed an ossified lesser horn of the hyoid on the right side and one circular ossification center in the thyroid cartilage (Fig. 4).

All pediatric hyoid-larynx complexes had a non-fused hyoid bone and normal greater horns (Fig. 3). Samples 1–4 contained bilateral cartilaginous lesser horns and no anomalies regarding the superior thyroid horns. The sample of the 16-year-old (case 5) exhibited a right-side ossified lesser horn, a left-side cartilaginous lesser horn, and bilateral triticeal cartilages. The sample of the 14-year-old (case 4) contained two bone fragments on the stylohyoid ligament. Additionally, samples 3 and 5 showed small circular defects in the lamina of the thyroid cartilage (Fig. 4).

### Staining and diceCT findings

Complete staining with B-Lugol was achieved in three specimens. These samples weighed 4–17 g and were completely stained in 4–5 days (Table 2). The largest pediatric sample weighed 121 g, and only the outer layers of the hyoid-larynx complex were stained after a staining time of 16 days. This resulted in stained bone and cartilage tissue, while the soft tissue in the center of the sample remained unstained.

**Fig. 5:**
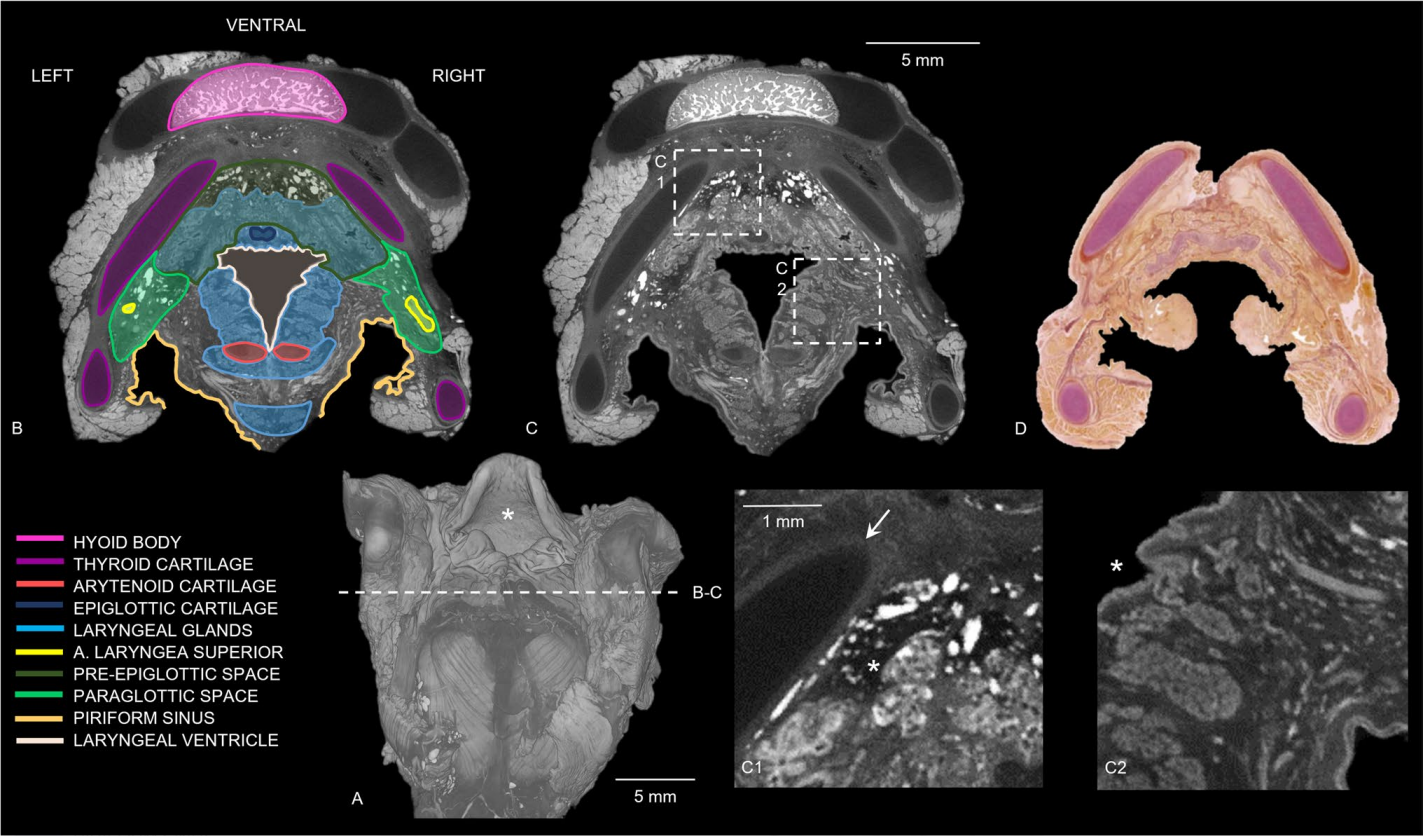
3D diceCT volume rendering and diceCT transverse sections of the sample of the 12-day-old female (case 1) (voxel size 12 μm), anatomically marked and compared with a histological section. (**a**) 3D diceCT volume rendering, dorsal view, showing the epiglottis (*asterisk*) and section of (**b**)-(**c**). (**b**) DiceCT transverse slice with anatomically marked sections and locations. (**c**) Same transverse slice as (**a**); showing a hyperdense ossified hyoid body, hyperdense connective tissue in paraglottic and pre-epiglottic space and a denser line surrounding the hypodense cartilages. (c1) Section of image (**c**) showing the denser line around the more hypodense thyroid cartilage (*arrow*) and hyperdense connective tissue (*asterisk*) in the pre-epiglottic space. (c2) Section of image c showing individually visible hypodense glands surrounding the laryngeal ventricle (*asterisk*). (**d**) Transverse section of the human newborn larynx at 5 mm above the glottis with Elastica van Gieson stain, from “Functional Histoanatomy of the Human Larynx” [19] for comparison of the detailed diceCT image in (**c**). Note that the hyoid bone is not visualized in the histological section. Adapted with permission from Springer Nature Customer Service Centre GmbH: Springer Singapore, Functional Histoanatomy of the Human Larynx by Kiminori Sato. Copyright Springer Nature Singapore Pte Ltd. 2018

DiceCT revealed uni- or bilateral hyperdense bundles crossing through the thyroid lamina in samples 3 and 5 (Fig. 4). A hyperdense ossification center was identified within the thyroid cartilage in the oldest pediatric sample and in addition some hypodense aspects were found in the cricoid cartilage (Fig. 4). In all specimens, a line with higher density was noted encircling the hypodense cartilage parts of the hyoid (if not completely ossified) and larynx on diceCT (Figs. 4 and 5). Figure 5 shows a 3D diceCT volume rendering and anatomically marked transverse sections of sample 1 to show the detailed visualization of the pediatric hyoid-larynx complex’s anatomy.

### Standard forensic assessment

The total-body CT and autopsy reports revealed no traumatic findings. On micro-CT and diceCT, no fractures or hemorrhages were identified in any of the pediatric samples, which is consistent with the total-body CT and autopsy findings.

## Discussion

Our aim was to evaluate the applicability of micro-CT and diceCT imaging for the detailed visualization of the pediatric hyoid-larynx complex. Traditional forensic imaging modalities face limitations in pediatric cases due to the non-calcified nature of the hyoid-larynx complex, limited resolution, and lack of contrast [3, 4]. In this study, we demonstrate that micro-CT and diceCT overcome these challenges by providing high-resolution and soft tissue contrast.

### Anatomy and embryology

All pediatric specimens demonstrated a non-fused ossified hyoid bone with either bilateral or unilateral cartilaginous lesser horns (Fig. 3). These findings align with known ossification patterns of the hyoid and laryngeal structures, as hyoid ossification starts within the first two living years and calcification of the larynx does not start before the second decade [2]. In addition, literature reports that the calcification of the larynx starts within the inferior thyroid horns and spreads to the superior thyroid horns [2, 4]. Nevertheless, our results show a sample of a 16-year-old male that already showed one calcified center in the thyroid lamina as the beginning of ossification (Fig. 4). This center was identified as an ossification center as Claassen et al. [20] found that the laryngeal ossification pattern consists of a special mode of endochondral ossification, which includes the mineralization of cartilage and creating islands of cartilage before being covered by the deposition of bone. The hyperdense center could represent the mineralized cartilage, as this cartilage appears denser than bone on X-ray images [20].

Furthermore, hypodense features were identified within the cartilages of the hyoid-larynx complex of this 16-year-old (Fig. 4). These features could present part of the ossification process. Dedivitis et al. [21] studied histological aging changes in the cricoid cartilage and showed that adolescents’ cricoid cartilages only consist of typical hyaline cartilage, while the older cartilages showed central areas of lamellar bone tissue and bone marrow cavities filled with adipose and/or hematopoietic tissue. These cavities could possibly be seen as hypodense irregularities in the larynx cartilages on micro-CT.

In addition, a denser layer was observed around the hypodense cartilages in all pediatric samples (Fig. 4). This layer appeared as a hypodense line surrounding the even more hypodense laryngeal cartilages, and in the younger samples, it also encircled the hypodense cartilaginous greater horns of the hyoid (Fig. 5). This could shed light on the cartilage and bone development, as it was not observed in adults [14]. The dense layer could present the outer layer of hyaline cartilage, the perichondrium. With age, the cartilage is subject to the process of endochondral ossification, resulting in the perichondrium being converted into periosteum [20]. Periosteum is a thin layer of membranous connective tissue, while perichondrium is denser and consists of fibrous tissue [22]. Besides, the perichondrium appears thinner on histological slices of the cartilaginous adult larynx in comparison with the pediatric larynx; therefore, it could show less dense on diceCT as well [19].

In two samples, a uni- or bilateral circular defect in the lamina of the thyroid cartilage was observed on micro-CT (Fig. 4). In the literature, this opening is known as the foramen thyroideum (FT) [23]. The FT can occur in one or both laminae of the thyroid cartilage. We found a FT in two of the hyoid-larynx complexes (40%), in one sample unilateral and in the other one bilateral. Previous research reports an incidence of FT in adults of 24–31%, of which 2–11% were found bilaterally [23–25]. The prevalences found in this study showed a slight difference in comparison to current literature, which could be due to our small sample size. According to León et al. [25], the adult FT contains a neurovascular bundle in 73%, a nerve branch in 20%, and in 7% only a vascular branch. In our research, diceCT showed a hyperdense bundle within every FT. Within those hyperdense bundles, two hypodense tubular structures could be visualized in one sample, and the other sample showed one hypodense and one hyperdense tubular structure (Fig. 4). Since blood shows hyperdense on diceCT images, we suspect that the hypodense tubular structures are nerves (branch of the superior laryngeal nerve) and the hyperdense structures are blood-filled vascular branches. As neurovascular bundles are surrounded by connective tissue, the hyperdense staining surrounding the bundles is considered connective tissue. This is in line with the hyperdensely stained tissue we discovered in the cricoid area, paraglottic, and pre-epiglottic spaces (Fig. 5). These spaces contain areolar or loose connective tissue, which is composed of adipose tissue with elastic and collagen fibers [22]. Hence, B-Lugol binds to adipose tissue and/or elastic and collagen fibers as well. Anatomical dissection could provide a definitive answer to the content of the FT.

The observed ossification process, hyperdensely stained perichondrium, and FT provide potential new insights into early hyoid-larynx complex development and anatomy, highlighting the potential of micro-CT and diceCT for developmental anatomy research.

### Forensic implications

The forensic relevance of these imaging techniques lies in their ability to non-destructively assess the pediatric hyoid-larynx complex in micrometer-level detail, something previously unattainable using standard imaging or autopsy methods. Partial ossifications and transitional zones, which are difficult or impossible to assess macroscopically, become clearly visible. In contrast to traditional histology, diceCT enables three-dimensional mapping of hemorrhages in situ and in anatomical context, without requiring destructive tissue dissection [20]. This allows for more precise, targeted histological sampling and preserves the integrity of delicate pediatric structures. Second, the high resolution of micro-CT and diceCT makes it possible to identify subtle trauma and visualize hematomas with confidence [20]. Given this level of detail, a negative scan may reasonably exclude significant hyoid-larynx trauma. With advancing technology and growing interest, micro-CT and diceCT may in time become valuable tools for routine forensic evaluation. Importantly, current forensic protocols do not recommend separate imaging of the excised pediatric larynx and hyoid, largely due to the insufficient resolution of conventional modalities. Now that micro-CT offers a viable alternative, there is a strong case for systematic research into its forensic value, with the potential to revise and improve existing guidelines. Third, the ability to clearly visualize ossification centers and perichondral layers could support forensic age estimation. Finally, the resulting 3D volume renderings are not only scientifically informative but also provide clear and compelling visualizations for courtroom presentations. Compared to traditional autopsy photos or histological slides, these high-resolution images enhance communication of complex anatomical findings to legal professionals and lay audiences alike.

### Limitations

This study’s small sample size limits the generalizability of the findings. Additionally, incomplete staining in the largest pediatric sample highlights the need for optimized staining protocols. In hindsight, this sample contained a lot of soft tissue and, based on its weight compared to the other samples, required a longer staining time. Based on our experience, we estimate that this particular sample would have required approximately 30 days of staining to achieve complete and uniform contrast enhancement. To strengthen the forensic applicability of diceCT, future research should include trauma cases and incorporate histological validation to confirm the accuracy of imaging findings. Finally, some practical limitations of micro-CT should be acknowledged: the technique requires access to high-end imaging infrastructure, is associated with significant costs, and demands specialized expertise for both image acquisition and interpretation. These factors may currently limit its widespread adoption in routine forensic workflows, particularly outside academic or research-oriented settings.

## Conclusion

We have shown that Micro-CT and diceCT provide high-resolution imaging of the pediatric hyoid-larynx complex and overcome the challenges of conventional forensic imaging modalities. These techniques enable detailed anatomical visualization, enhancing forensic investigations of pediatric neck trauma. Future research should focus on including trauma cases and validating findings against histological data to fully establish the forensic applicability and value of these imaging methods.

## Data Availability

No datasets were generated or analysed during the current study.

## References

[1] Ubelaker DH (1992) Hyoid fracture and strangulation. J Forensic Sci 37:1216–1222 1402747

[2] Pollanen MS, Ubelaker DH (1997) Forensic significance of the polymorphism of hyoid bone shape. J Forensic Sci 42:890–892 9304837

[3] de Bakker BS, de Bakker HM, Soerdjbalie-Maikoe V, Dikkers FG (2019) Variants of the hyoid-larynx complex, with implications for forensic science and consequence for the diagnosis of Eagle’s syndrome. Sci Rep 9:15950 31685955 10.1038/s41598-019-52476-z PMC6828966

[4] Hudgins PA, Siegel J, Jacobs I, Abramowsky CR (1997) The normal pediatric larynx on CT and MR. AJNR Am J Neuroradiol 18(2):239–45 PMC8338579 9111658

[5] de Bakker HM, Olsthoorn PC, Soerdjbalie-Maikoe V, de Bakker BS (2020) Comparison of post-mortem radiologic modalities to evaluate suspected neck violence. Forensic Imaging 21:200373

[6] Gascho D, Heimer J, Tappero C, Schaerli S (2019) Relevant findings on postmortem CT and postmortem MRI in hanging, ligature strangulation and manual strangulation and their additional value compared to autopsy - a systematic review. Forensic Sci Med Pathol 15:84–92 30627977 10.1007/s12024-018-0070-z

[7] Treitl KM, Aigner LI, Gazov E et al (2020) Injuries of the isolated larynx-hyoid complex in post-mortem computed tomography (PMCT) and post-mortem fine preparation (PMFP) - a comparison of 54 forensic cases. Eur Radiol 30:4564–4572 32232789 10.1007/s00330-020-06770-4 PMC8275497

[8] Dawood Y, Buijtendijk MFJ, Shah H et al (2022) Imaging fetal anatomy. Semin Cell Dev Biol 131:78–92 35282997 10.1016/j.semcdb.2022.02.023

[9] Hutchinson JC, Shelmerdine SC, Simcock IC et al (2017) Early clinical applications for imaging at microscopic detail: microfocus computed tomography (micro-CT). Br J Radiol 90:20170113 28368658 10.1259/bjr.20170113 PMC5594989

[10] Pelberg RA, Mazur W (2011) CT Basics. Vascular CT angiography manual. Springer London, London, pp 3–24

[11] Gignac PM, Kley NJ, Clarke JA et al (2016) Diffusible iodine-based contrast-enhanced computed tomography (diceCT): an emerging tool for rapid, high-resolution, 3-D imaging of metazoan soft tissues. J Anat 228:889–909 26970556 10.1111/joa.12449 PMC5341577

[12] Degenhardt K, Wright AC, Horng D et al (2010) Rapid 3D phenotyping of cardiovascular development in mouse embryos by micro-CT with iodine staining. Circ Cardiovasc Imaging 3(3):314–322 20190279 10.1161/CIRCIMAGING.109.918482 PMC3059892

[13] Paulis MG, Ali DM (2018) Antemortem, perimortem and postmortem bone fracture: could histopathology differentiate between them? Egypt J Forensic Sci Appl Toxicol 18:135–160

[14] Timmerman GMM, van Goethem A, Docter D et al (2025) The value of micro-CT imaging in the forensic evaluation of neck trauma. Forensic Sci Int 374:112544 10.1016/j.forsciint.2025.112544 40578128

[15] Di Nunno N, Lombardo S, Costantinides F, Di Nunno C (2004) Anomalies and alterations of the hyoid-larynx complex in forensic radiographic studies. Am J Forensic Med Pathol 25:14–19 15075682 10.1097/01.paf.0000113931.49721.e4

[16] Soerdjbalie-Maikoe V, van Rijn RR (2008) Embryology, normal anatomy, and imaging techniques of the hyoid and larynx with respect to forensic purposes: a review article. Forensic Sci Med Pathol 4:132–139 19291485 10.1007/s12024-008-9032-1

[17] Mupparapu M, Vuppalapati A (2005) Ossification of laryngeal cartilages on lateral cephalometric radiographs. Angle Orthod 75:196–201 15825782 10.1043/0003-3219(2005)075<0192:OOLCOL>2.0.CO;2

[18] Dawood Y, Hagoort J, Siadari BA et al (2021) Reducing soft-tissue shrinkage artefacts caused by staining with Lugol’s solution. Sci Rep 11:19781 34611247 10.1038/s41598-021-99202-2 PMC8492742

[19] Sato K (2018) Functional histoanatomy of the human larynx. Springer Singapore

[20] Claassen H, Schicht M, Sel S, Paulsen F (2014) Special pattern of endochondral ossification in human laryngeal cartilages: X-ray and light-microscopic studies on thyroid cartilage. Clin Anat 27:423–430 24496984 10.1002/ca.22309

[21] Dedivitis R, Abrahão M, Simoes M et al (2004) Aging histological changes in the cartilages of the cricoarytenoid joint. Acta Cirurgica Brasileira 19. 10.1590/S0102-86502004000200010

[22] Samanthi U. Difference between perichondrium and periosteum. DifferenceBetween.com. https://www.differencebetween.com/difference-between-perichondrium-and-periosteum/. Accessed 16 Dec 2022

[23] Menasinkai SB, Savitha V (2015) A study of foramina thyroideum. Int J Anat Res 3:1152–5

[24] Raikos A, Paraskevas GK (2013) The thyroid foramen: a systematic review and surgical considerations. Clin Anat 26:700–708 23553826 10.1002/ca.22234

[25] León X, Maranillo E, Mirapeix RM et al (1997) Foramen thyroideum: a comparative study in embryos, fetuses, and adults. Laryngoscope 107:1146–1150 9261024 10.1097/00005537-199708000-00026

